# Manthan: A Data-Driven Approach for Boolean Function Synthesis

**DOI:** 10.1007/978-3-030-53291-8_31

**Published:** 2020-06-16

**Authors:** Priyanka Golia, Subhajit Roy, Kuldeep S. Meel

**Affiliations:** 8 Microsoft Research Lab, Redmond, WA USA; 9 University of Southern California, Los Angeles, CA USA; 10 Computer Science and Engineering, Indian Institute of Technology Kanpur, Kanpur, India; 11 School of Computing, National University of Singapore, Singapore, Singapore

## Abstract

Boolean functional synthesis is a fundamental problem in computer science with wide-ranging applications and has witnessed a surge of interest resulting in progressively improved techniques over the past decade. Despite intense algorithmic development, a large number of problems remain beyond the reach of the state of the art techniques.

Motivated by the progress in machine learning, we propose $\textsf {Manthan}$, a novel data-driven approach to Boolean functional synthesis. $\textsf {Manthan}$ views functional synthesis as a classification problem, relying on advances in constrained sampling for data generation, and advances in automated reasoning for a novel proof-guided refinement and provable verification. On an extensive and rigorous evaluation over 609 benchmarks, we demonstrate that $\textsf {Manthan}$ significantly improves upon the current state of the art, solving 356 benchmarks in comparison to 280, which is the most solved by a state of the art technique; thereby, we demonstrate an increase of 76 benchmarks over the current state of the art. Furthermore, $\textsf {Manthan}$ solves 60 benchmarks that none of the current state of the art techniques could solve. The significant performance improvements, along with our detailed analysis, highlights several interesting avenues of future work at the intersection of machine learning, constrained sampling, and automated reasoning.

## Introduction

Given an existentially quantified Boolean formula $\exists Y F(X,Y)$ over the set of variables *X* and *Y*, the problem of Boolean functional synthesis is to compute a vector of Boolean functions, denoted by $\varPsi (X) = \langle \psi _{1}(X), \psi _{2}(X), \ldots ,\psi _{|Y|}(X)\rangle$, and referred to as Skolem function vector, such that $\exists Y F(X,Y) \equiv F(X, \varPsi (X))$. In the context of applications, the sets *X* and *Y* are viewed as inputs and outputs, and the formula *F*(*X*, *Y*) is viewed as a functional specification capturing the relationship between *X* and *Y*, while the Skolem function vector $\varPsi (X)$ allows one to determine the value of *Y* for the given *X* by evaluating $\varPsi$. The study of Boolean functional synthesis traces back to Boole [12], and over the decades, the problem has found applications in a wide variety of domains such as certified QBF solving [8, 9, 36, 41], automated program repair [27], program synthesis [44], and cryptography [35].

Theoretical investigations have demonstrated that there exist instances where Boolean functional synthesis takes super-polynomial time. On the other hand, practical applicability has necessitated the development of algorithms with progressively impressive scaling. The algorithmic progress for Boolean functional synthesis has been driven by a diverse set of techniques: (i) the usage of incremental determinization employing the several heuristics in state-of-the-art Conflict Driven Clause Learning (CDCL) solvers [41], (ii) usage of decomposition techniques employing the progress in knowledge compilation [6, 19, 28, 45], and (iii) Counter-Example Guided Abstraction Refinement (CEGAR)-based techniques relying on usage of SAT solvers as black boxes [4–6, 28]. While the state of the art techniques are capable of handling problems of complexity beyond the capability of tools a decade ago, the design of scalable algorithms capable of handling industrial problems remains the holy grail.

In this work, we take a step towards the above goal by proposing a novel approach, called $\textsf {Manthan}$, at the intersection of machine learning, constrained sampling, and automated reasoning. Motivated by the unprecedented advances in machine learning, we view the problem of functional synthesis through the lens of multi-class classification aided by the generation of the data via constrained sampling and employ automated reasoning to certify and refine the learned functions. To this end, the architecture of $\textsf {Manthan}$ comprises of the following three novel techniques:

- **Data Generation.** The state of the art machine learning techniques use training data represented as a set of samples where each sample consists of valuations to features and the corresponding label. In our context, we treat *X* as the features and *Y* as labels. Unlike the standard setup of machine learning wherein for each assignment to *X*, there is a unique label, i.e. assignment to *Y*, the relationship between *X* and *Y* is captured by a relation and not necessarily a function. To this end, we design a weighted sampling strategy to generate a *representative* data set that can be fitted using a *compactly sized* classifier. The weighted sampling strategy, implemented using state of the constrained sampler, seeks to uniformly sample input variables (*X*) while biasing the valuations of output variables towards a particular value.
- **Dependency-Driven Classifier for Candidates.** Given training data viewed as a valuation of *features* (X) and their corresponding labels (Y), a natural approach from machine learning perspective would be to perform multi-class classification to obtain $Y = h (X)$, where *h* is a symbolic representation of the learned classifier. Such an approach, however, can not ensure that *h* can be expressed as a vector of Boolean functions. To this end, we design a dependency aware classifier to construct a vector of decision trees corresponding to each $Y_i$, wherein each decision tree is expressed as a Boolean function.
- **Proof-Guided Refinement.** Since machine learning techniques often produce good but inexact approximations, we augment our method with automated reasoning techniques to verify the correctness of decision tree-based candidate Skolem functions. To this end, we perform a counterexample driven refinement approach for candidate Skolem functions.
  To fully utilize the impressive test accuracy attained by machine learning models, we design a *proof-guided refinement* approach that seeks to identify and apply *minor* repairs to the candidate functions, in an iterative manner, until we converge to a provably correct Skolem function vector. In a departure from prior approaches utilizing the Shannon expansion and self-substitution, we first use a MaxSAT solver to determine potential repair candidates, and employ unsatisfiability cores obtained from the infeasibility proofs capturing the reason for current candidate functions to meet the specification, to construct a *good repair*.

Finally, We perform an extensive evaluation over a diverse set of benchmarks with state-of-the-art tools, viz. C2Syn [4], BFSS [5], and CADET [39]. Of 609 benchmarks, $\textsf {Manthan}$ is able to solve 356 benchmarks while C2Syn, BFSS, and CADET solve 206, 247, and 280 benchmarks respectively. Significantly, $\textsf {Manthan}$ can solve 60 benchmarks beyond the reach of all the other existing tools extending the reach of functional synthesis tools. We then perform an extensive empirical evaluation to understand the impact of different design choices on the performance of $\textsf {Manthan}$. Our study reveals several surprising observations arising from the inter-play of machine learning and automated reasoning.

$\textsf {Manthan}$ owes its runtime performance to recent advances in machine learning, constrained sampling, and automated reasoning. Encouraged by $\textsf {Manthan}$’s scalability, we will seek to extend the above approach to related problem domains such as automated program synthesis, program repair, and reactive synthesis.

The rest of the paper is organized as follows: We first introduce notations and preliminaries in Sect. 2. We then discuss the related work in Sect. 3. In Sect. 4 we present an overview of $\textsf {Manthan}$ and give an algorithmic description in Section  5. We then describe the experimental methodology and discuss results in Sect. 6. Finally, we conclude in Sect. 7.

## Notations and Preliminaries

We use lower case letters (with subscripts) to denote propositional variables and upper case letters to denote a subset of variables. The formula $\exists Y F(X,Y)$ is existentially quantified in *Y*, where $X=\{x_1,\cdots ,x_n\}$ and $Y=\{y_1,\cdots ,y_m\}$. For notational clarity, we use *F* to refer to *F*(*X*, *Y*) when clear from the context. We denote *Vars*(*F*) as the set of variables appearing in *F*(*X*, *Y*). A literal is a boolean variable or its negation. We often abbreviate universally (resp. existentially) quantified variables as universal (resp. existential) variables.

A *satisfying assignment* of a formula *F*(*X*, *Y*) is a mapping $\sigma : Vars(F) \rightarrow \{0,1\}$, on which the formula evaluates to True. For $V \subseteq Vars(F)$, $\sigma [V]$ represents the truth values of variables in *V* in a satisfying assignment $\sigma$ of *F*. We denote the set of all witnesses of *F* as $R_{F}$. For a formula in conjunctive normal form, the *unsatisfiable core*(UnsatCore) is a subset of clauses of the formula for which no satisfying assignment exists.

We use $F(X,Y)|_{y_i=b}$ to denote *substitutions*: a formula obtained after substituting every occurrence of $y_i$ in *F*(*X*, *Y*) by *b*, where *b* can be a constant (0 or 1) or a formula. The operator *ite(condition,exp1,exp2)* is used to represent the if-else case: if the *condition* is true, then it returns *exp1*, else it returns *exp2*.

A variable $y_i$ is considered as a *positive unate* if and only if $F(X,Y)|_{y_i=0} \wedge \lnot F(X,Y)|_{y_i=1}$ is UNSAT and a *negative unate* if and only if $F(X,Y)|_{y_i=1} \wedge \lnot F(X,Y)|_{y_i=0}$ is UNSAT [5].

Given a function vector $\langle \psi _1,\ldots ,\psi _m \rangle$ for the vector of variables $\langle y_1, \ldots y_m \rangle$ such that $\psi _i$ is the function corresponding to $y_i$, we say that there exists a partial order $\prec _{d}$ over the variables $\{y_1, \ldots y_m\}$ such that $y_i \prec _{d} y_j$ if $\psi _i$ depends on $y_j$.

In decision tree learning, a fraction of incorrectly assigned labels refer to the *impurity*. We use Gini Index [38] as a measure of *impurity* for a class label. The *impurity decrease* at a node is the difference of its impurity to the mean of impurities of its children. The *minimum impurity decrease* is a hyper-parameter used to control the maximum allowable impurity at the leaf nodes, thereby providing a lever for how closely the classifier fits the training data.

Given a propositional formula *F*(*X*, *Y*) and a weight function $W(\cdot )$ assigning non-negative weights to every literal, we refer to the *weight* of a satisfying assignment $\sigma$, denoted as $W(\sigma )$, as the product of weights of all the literals appearing in $\sigma$, i.e., $W(\sigma ) = \prod _{l \in \sigma } W(l)$. A *sampler* $\mathcal {A}(\cdot , \cdot )$ is a probabilistic generator that guarantees $\forall \sigma \in R_{F},$ $\textsf {Pr}\left[ \mathcal {A}(F, \textsf {Bias}) = \sigma \right] \propto W(\sigma )$.

We use a function $\textsf {Bias}$ that takes a mapping from a sequence of variables to the desired weights of their positive literals, and assigns corresponding weights to each of the positive literals. We use a simpler notation, $\textsf {Bias}$(a,b) to denote that positive literals corresponding to all universal variables are assigned a weight a and positive literals corresponding to all existential variables are assigned a weight b. For example, $\textsf {Bias}$(0.5, 0.9) assigns a weight of 0.5 to the positive literals of the universally quantified variables and 0.9 to the positive literals of the existentially quantified variables.

***Problem Statement:*** Given a Boolean specification *F*(*X*, *Y*) between set of inputs $X = \{x_1,\cdots ,x_n\}$ and vector of outputs $Y=\langle y_1,\cdots ,y_m\rangle$, the problem of *Skolem function synthesis* is to synthesize a function vector $\varPsi = \langle \psi _1(X),\cdots , \psi _m(X)\rangle$ such that $y_i \leftrightarrow \psi _i(X)$ and $\exists Y F(X,Y) \equiv F(X,\varPsi )$. We refer to $\varPsi$ as the *Skolem function vector* and $\psi _i$ as the *Skolem function* for $y_i$.

A variable $y_i$ is called self-substituted variable, if the Skolem function $\psi _i$ corresponding to $y_i$ is set to $F(X,Y)|_{y_i=1}$ [19].

Given a formula $\exists Y F(X,Y)$ and a Skolem function vector $\varPsi$, we refer to $E(X,Y,Y')$ as an *error formula* [28], where $Y'=\{y'_1,\cdots ,y'_{|Y|}\}$, and $Y' \ne Y$.

1 $$\begin{aligned} E(X,Y,Y') = F(X,Y) \wedge \lnot F(X,Y') \wedge (Y'\leftrightarrow \varPsi ) \end{aligned}$$

We use the following theorems from prior work:

### Theorem 1

**(**[28]**).** $\varPsi$ is a Skolem function if and only if $E(X,Y,Y')$ is UNSAT.

### Theorem 2

**(**[5]**).** If $y_i$ is positive(*resp* negative) unate in *F*(*X*, *Y*), then $\psi _i = 1$ (*resp* $\psi _i = 0$) is the Skolem function for $y_i$.

## Related Work

The origins of the problem of Boolean functional synthesis traces back to Boole’s seminal work [12], which was subsequently rigorously pursued, albeit focused on decidability, by Lowenheim and Skolem [33]. The complexity theoretic studies have shown that there exist instances where Boolean functional synthesis takes super polynomial time and was also shown that there exist instances for which polynomial size Skolem function vector does not suffice unless Polynomial Hierarchy (PH) collapses [5].

Motivated by the success of the CEGAR (Counter-Example Guided Abstraction Refinement) approach in model checking, CEGAR-based approaches have been pursued in the context of synthesis as well, where the key idea is to use a Conflict-Driven Clause Learning (CDCL) SAT solver to verify and refine the candidate Skolem functions [4–6, 28].

Another line of work has focused on the representation of specification, i.e., *F*(*X*, *Y*), in representations that are amenable to efficient synthesis for a class of functions. The early approaches focused on ROBDD representation building on the functional composition approach proposed by Balabanov and Jiang [8]. Building on Tabajara and Vardi’s ROBDD-based approach [45], Chakraborty et al. extended the approach to factored specifications [14]. It is worth mentioning that factored specifications had earlier been pursued in the context of CEGAR-based approaches. Motivated by the success of knowledge compilation in the field of probabilistic reasoning, Akshay et al. achieved a significant breakthrough over a series of papers [5, 6, 28] to propose a new negation normal form, SynNNF [4]. The generalization and a functional specification presented in SynNNF is amenable to efficient functional synthesis [4]. Another line of work focused on the usage of *incremental determinization* to incrementally construct the Skolem functions [25, 30, 36, 39, 41].

Several approaches have been proposed for the particular case when the specification, $\exists Y F(X,Y)$ is valid, i.e., $\forall X \exists Y F(X,Y)$ is True. Inspired by the sequential relational decomposition, Chakraborty et al. [14] recently proposed an approach focused on viewing each CNF clause of the specification consisting of *input and output* clauses and employing a *cooperation*-based strategy. The progress in modern CDCL solvers has led to an exploration of usage of heuristics for problems in complexity classes beyond NP. This has led to work on the extraction of Skolem functions from the proofs constructed for the formulas expressed as $\forall X \exists Y F(X,Y)$ [8, 9].

The performance of Manthan crucially depends on its ability to employ constrained sampling, which has witnessed a surge of interest with approaches ranging from those based on hashing-based techniques [15], knowledge compilation [24, 42], augmentation of SAT solvers with heuristics [43].

The recent success of machine learning has led to several attempts to the usage of machine learning in several related synthesis domains such as program synthesis [7], invariant generation, decision-tree for functions in Linear Integer Arithmetic theory using pre-specified examples [18], strategy synthesis for QBF [26]. Use of data-driven approaches for invariant synthesis has been investigated in the ICE learning framework [17, 20, 21] aimed with data about the program behavior from test executions, it proposes invariants by learning from data, checks for inductiveness and, on failure, extend the data by the generated counterexamples. The usage of proof-artifacts such as unsat cores has been explored in verification since early 2000s [23] and in program repair in Wolverine [46], while MaxSAT has been used in program debugging in [10, 29].

## $\textsf {Manthan}$: An overview

**Fig. 1.**
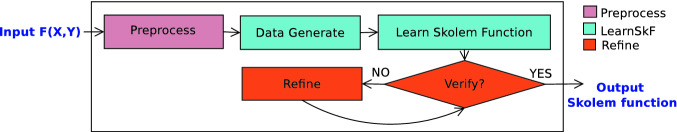
Overview of $$\textsf {Manthan}$$

In this section, we provide an overview of our proposed framework, $\textsf {Manthan}$, before divulging into core algorithmic details in the following section. $\textsf {Manthan}$ takes in a function specification, represented as *F*(*X*, *Y*), and returns a Skolem function vector $\varPsi (X)$ such that $\exists Y F(X,Y)$ $\equiv$ $F(X,\varPsi (X))$. As shown in Fig. 1 $\textsf {Manthan}$ consists of following three phases:

1. $\textsf {Preprocess}$ employs state-of-the-art pre-processing techniques on *F* to compute a partial Skolem function vector.
2. $\textsf {LearnSkF}$ takes in the pre-processed formula and uses constrained samplers, and classification techniques to compute candidate Skolem functions for all the variables in *Y*.
3. $\textsf {Refine}$ performs verification and proof-guided refinement procedure wherein a SAT solver is employed to verify the correctness of candidate functions and a MaxSAT solver in conjunction with a SAT solver is employed to refine the candidate functions until the entire candidate Skolem function vector passes the verification check.

We now provide a high-level description of different phases to highlight the technical challenges, which provides context for several algorithmic design choices presented in the next section.

### Phase 1: $\textsf {Preprocess}$

$\textsf {Preprocess}$ focuses on pre-processing of the formula to search for unates among the variables in *Y*; if $y_i$ is positive (resp. negative) unate, then $\psi _i = 1$(resp. 0) suffices as a Skolem function. We employ the algorithmic routine proposed by Akshay et al. [5] to drive this preprocessing.

### Phase 2: LearnSkF

$\textsf {LearnSkF}$ views the problem of functional synthesis through the lens of machine learning where the learned machine learning model for classification of a variable $y_i$ can be viewed as a candidate Skolem function for $y_i$. We gather training data about the function’s behavior by exploiting the progress in constrained sampling to sample solutions of *F*(*X*, *Y*). Recall that *F*(*X*, *Y*) defines a relation (and not necessarily a function) between *X* and *Y*, and the machine learning techniques typically assume the existence of function between features and labels, necessitating the need for sophisticated sampling strategy as discussed below. Moving on to features and labels, since we want to learn *Y* in terms of *X*, we view *X* as a set of features while assignments to *Y* as a set of class labels.

The off-the-shelf classification techniques typically require that the size of training data is several times larger than the size of possible class labels, which would be prohibitively large for the typical problems involving more than thousand variables. To mitigate the requirement of large training data, we make note of two well-known observations in functional synthesis literature: (1) the Skolem function $\psi _i$ for a variable $y_i$ typically does not depend on all the variables in *X*, (2) A Skolem function vector $\varPsi$ where $\psi _i$ depends on variable $y_j$ is a valid vector if the Skolem function $\psi _j$ is not dependent on $y_i$ (i.e., acyclic dependency), i.e., there exists a partial order $\prec _{d}$ over $\{y_1, \ldots y_m\}$.

The above observations lead us to design an algorithmic procedure where we learn candidate Skolem functions as decision trees in an iterative manner, i.e., one $y_i$ at a time, thereby allowing us to constrain ourselves to the binary classification. The learned classifier can then be represented as the disjunction of all the paths from the root to the leaves in the learnt decision tree. We update the set of possible features for a given $y_i$ depending on the candidate functions generated so far, i.e., valuation of *X* variables and *Y* variables, which are not dependent on $y_i$. Finally, we compute the candidate Skolem function for $y_i$ as the disjunction of labels along edges for all the paths from the root to leaf nodes with label 1. Once, we have the candidate Skolem function vector $\varPsi$, we obtain a valid linear extension, *TotalOrder*, of the partial order $\prec _{d}$ in accordance to $\varPsi$.

Before moving on to the next phase, we return to the formulation of sampling. The past few years have witnessed the design of uniform [15, 42], and weighted samplers [24], and one wonders what kind of sampler should we choose to generate samples for training data. A straightforward choice would be to perform uniform sampling over *X* and *Y*, but the relational nature of specification, *F*, between *X* and *Y* offers interesting challenges and opportunities. Recall while *F* specifies a relation between *X* and *Y*, we are interested in a Skolem function, and we would like to tailor our sampling subroutines to allow discovery of Skolem functions with *small* description given the relationship between description and sample complexity. To this end, consider $X = \{x_1, x_2\}$ and $Y= \{ y_1\}$, and let $F:= (x_1 \vee x_2 \vee y_1)$. Note that *F* has 7 solutions over $X \cup Y$, out of which $y_1=0$ appears in 3 solutions while $y_1 = 1$ appears in 4. Also, note that there are several possible Skolem functions such as $y_1 = \lnot (x_1 \wedge x_2)$. Now, if we uniformly sample solutions of *F* over ${x_1, x_2, y_1}$, i.e. $\textsf {Bias}$(0.5, 0.5), we would see (almost) equal number of samples with $y_1 = 0$ and $y_1 = 1$. A closer look at *F* reveals that it is possible to construct a Skolem function by knowing that the only case where $y_1$ cannot be assigned 0 is when $x_1 = x_2 =0$. To encode this intuition, we propose a novel idea of collecting samples with weighted sampling, i.e., $\textsf {Bias}$(0.5, q) where *q* is chosen in a multi-step process of first drawing a small set of samples with both $q = 0.9$ and $q=0.1$, and then drawing rest of the samples by fixing the value of *q* following analysis of an initial set of samples. To the best of our knowledge, this is the first application of weighted sampling in the context of synthesis, and our experimental results point to several interesting avenues of future work.

### Phase 3: Refine

The candidate Skolem functions generated in $\textsf {LearnSkF}$ may not always be the actual Skolem functions. Hence, we require a *verification* check to see if candidate Skolem functions are indeed correct; if not, the generated counterexample can be used to *repair* it. The verification query constructs an *error formula* $E(X,Y,Y')$ (Formula 1): if unsatisfiable, the candidate Skolem function vector is indeed a Skolem function vector and the procedure can terminate; else, when $E(X,Y,Y')$ is SAT, the solution of $E(X,Y,Y')$ is used to identify and refine the erring functions among the candidate Skolem function vector.

In contrast to prior techniques that apply Shannon expansion or self-substitution, the refinement strategy in $\textsf {Manthan}$ is guided by the view that the candidate function vector from the $\textsf {LearnSkF}$ phase is *almost correct*, and hence, attempts to identify and apply a series of *minor* repairs to the erring functions to arrive at the correct Skolem function vector. To this end, $\textsf {Manthan}$ uses two key techniques: *fault localization* and *repair synthesis*. Let us assume that $\sigma$ is a satisfying assignment of $E(X,Y,Y')$ and referred to as counterexample for the current candidate Skolem function vector $\varPsi$.

***Fault Localization.*** In order to identify the initial candidates to repair for the counterexample $\sigma$, $\textsf {Manthan}$ attempts to identify a small number of Skolem functions (correspondingly *Y* variables) whose outputs must undergo a change for the formula to behave correctly on $\sigma$; in other words, it makes a best-effort attempt to ensure that most of the Skolem functions (correspondingly *Y* variables) can retain their current output on $\sigma$ while satisfying the formula. $\textsf {Manthan}$ encodes this problem as a partial MaxSAT query with $F(X,Y) \wedge (X \leftrightarrow \sigma [X])$ as a hard constraint and $(Y \leftrightarrow \sigma [Y'])$ as soft constraints. All *Y* variables whose valuation constraint $(Y \leftrightarrow \sigma [Y'])$ does not hold in the MaxSAT solution are identified as erring Skolem functions that may need to be repaired.

***Repair Synthesis.*** Let $y_k$ be the variable corresponding to the erring function, $\psi _k$, identified in the previous step. To synthesize a repair for the function, $\textsf {Manthan}$ applies a proof-guided strategy: it constructs a formula $G_k(X,Y)$, such that if $G_k(X,Y)$ is unsatisfiable then $\psi _k$ must undergo a change. The UnsatCore of $G_k(X,Y)$ provides a *reason* that explains the discrepancy between the specification and the current Skolem function.

2 $$\begin{aligned}&\quad G_{k}(X,Y) = (y_k \leftrightarrow \sigma [y'_{k}]) \wedge F(X,Y) \wedge (X \leftrightarrow \sigma [X]) \wedge (\hat{Y} \leftrightarrow \sigma [\hat{Y}]) \nonumber \\&\mathrm {where}\, \hat{Y} \subset Y \; \mathrm {and} \; \hat{Y} = \{ \textit{TotalOrder}[index(y_k)+1],\cdots ,\textit{TotalOrder}[|Y|]\} \ \; \end{aligned}$$

$\textsf {Manthan}$ uses the UnsatCore to constructs a *repair formula*, say $\beta$, as a conjunction over literals in the unsatisfiable core; if $\psi _k$ is *true* with the current valuation of *X* and $\hat{Y}$, $\textsf {Manthan}$ updates the function $\psi _k$ by conjoining it with the negation of repair formula ($\psi _k \leftarrow \psi _k \wedge \lnot \beta$); otherwise, $\textsf {Manthan}$ updates the function $\psi _k$, by disjoining it with the repair formula ($\psi _k \leftarrow \psi _k \vee \beta )$.

**Self-substitution for Poorly Learnt Functions.** Some Skolem functions are difficult to learn through data. In our implementation, the corresponding variables escape the $\textsf {LearnSkF}$ phase with poor candidate functions, thereby requiring a long sequence of incremental repairs for convergence. To handle such scenarios, we make the following observation: though synthesizing Skolem functions via self-substitution [19] can lead to an exponential blowup in the worst case, it is inexpensive if the number of variables synthesized via this technique is small. We use this observation to quickly synthesize a Skolem function for an erring variable if we detect its candidate function is poor (detected by comparing the number of times it enters refinement against an empirically determined threshold). Of course, this heuristic does not scale well if the number of such variables is large; in our experiments, we found less than 20% of the instances solved required self-substitution, and for over 75% of these instances, only one variable needed self-substitution. We elaborate more on the empirical evidence on the success of this heuristic in Sect. 6. A theoretical understanding of the learnability of Boolean functions from data seems to be an interesting direction for future work.

## $\textsf {Manthan}$: Algorithmic Description


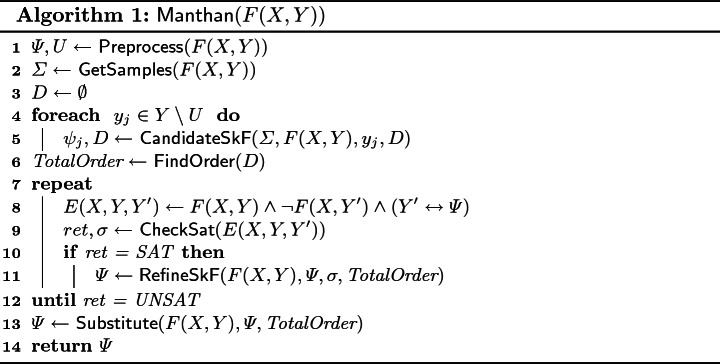


In this section, we present a detailed algorithmic description of $\textsf {Manthan}$, whose pseudocode is presented in Algorithm 1. $\textsf {Manthan}$ takes in a formula *F*(*X*, *Y*) as input and returns a Skolem vector $\varPsi$. The algorithm starts off by preprocessing (line 1) the formula *F*(*X*, *Y*) to get the unates (*U*) and their corresponding Skolem functions ($\varPsi$). Next, it invokes the sampler (line 2) to collect a set of samples$(\varSigma )$ as training data for the learning phase.

For each of the existential variables that are not unates, $\textsf {Manthan}$ attempts to learn candidate Skolem functions (lines 4–5). To generate a variable order, $\textsf {CandidateSkF}$ uses a collection of sets $d_1,\cdots ,d_{|Y|} \in D$, such that $y_i \in d_j$ indicates that $y_j$ depends on $y_i$. Next, the $\textsf {FindOrder}$ routine (line 6) construct *TotalOrder* of the *Y* variables in accordance to the dependencies in *D*. The verification and refinement phase (line 8) commences by constructing the error formula and launching the verification check (line 9). If the error formula is satisfiable, the counterexample model ($\sigma$) is used to refine the formula. Once the verification check is successful, the refinement phase ends and the subroutine $\textsf {Substitute}$ is invoked to recursively substitute all $y_i \in Y$ appearing in Skolem functions with their corresponding Skolem functions such that only *X* variables entirely describe all Skolem functions. The strict variable ordering enforced above ensures that $\textsf {Substitute}$ always succeeds and does not get stuck in a cycle. Finally, the Skolem function vector $\varPsi$ is returned.

It is worth noting that $\textsf {Manthan}$ can successfully solve an instance without having to necessarily execute all the phases. In particular, if $U = Y$, then $\textsf {Manthan}$ terminates after $\textsf {Preprocess}$ (i.e., line 1). Similarly, if the $\textsf {CheckSat}$ return UNSAT during the first iteration of loop (lines 8–11), then $\textsf {Manthan}$ does not invoke $\textsf {RefineSkF}$.

We now discuss each subroutine in detail. The pseudocode for $\textsf {Preprocess}$, $\textsf {GetSamples}$ and $\textsf {Substitute}$ is deferred to technical report [22].

$\textsf {Preprocess}$: We perform the pre-processing step as described in [5], which performs SAT queries on the formulas constructed as specified in Theorem 2.

$\textsf {GetSamples}$: $\textsf {GetSamples}$ takes *F*(*X*, *Y*) as input and returns a subset of satisfying assignments of *F*(*X*, *Y*). $\textsf {GetSamples}$ first generates a small set of samples (500) with $\textsf {Bias}$(0.5, 0.9) and calculates $m_i$ for all $y_i$, $m_i$ is a ratio of number of samples with $y_i$ being 1 to the total number of samples. Similarity, $\textsf {GetSamples}$ generates 500 samples with $\textsf {Bias}$(0.5, 0.1) and calculates $n_i$ for all $y_i$, $n_i$ is a ratio of number of samples with $y_i$ being 0 to the total number of samples. Finally, $\textsf {GetSamples}$ generates required number of samples with $\textsf {Bias}$(0.5, *q*); for a $y_i$, *q* is $m_i$ if both $m_i$ and $n_i$ are in range 0.35 to 0.65, else *q* is 0.9.
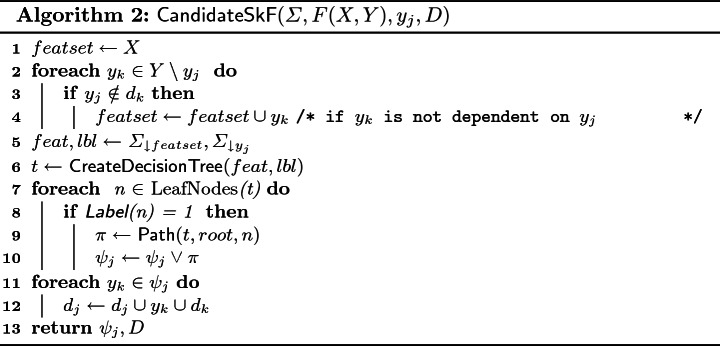


$\textsf {CandidateSkF}$: $\textsf {CandidateSkF}$, presented in Algorithm 2, assumes access to following three subroutines:

1. $\textsf {CreateDecisionTree}$ takes the feature and label sets as input (training data) and returns a decision tree *t*. We use the ID3 algorithm [38] to construct a decision tree *t* where the internal node of *t* represents a feature on which a decision is made, the branches represent partitioning of the training data on the decision, and the leaf nodes represent the classification outcomes (i.e class labels). The ID3 algorithm iterates over the training data, and in each iteration, it selects a new attribute to extend the tree by a new decision node: the selected attribute is one that causes the maximum drop in the impurity of the resulting classes; we use Gini Index [38] as the measure of impurity. The algorithm, then, extends the tree by the selected decision and continues extending building the tree. The algorithm terminates on a path if either it exhausts all attributes for decisions, or the impurity of the resulting classes drop below a (user-specified) impurity decrease parameter.
2. $\textsf {Label}$ takes a leaf node of the decision tree as input and returns the class label corresponding to the node.
3. $\textsf {Path}$ takes a tree *t* and two nodes of *t* (node *a* and node *b*) as input and outputs a conjunction of literals in the path from node *a* to node *b* in *t*.

As we seek to learn Boolean functions, we employ binary classifiers with class labels 0 and 1. $\textsf {CandidateSkF}$ shows our algorithm for extracting a Boolean function from the decision trees: lines 2–4 find a feature set (*featset*) to predict $y_j$. The feature set includes all *X* variables and the subset of *Y* variables that are not dependent on $y_j$. $\textsf {CandidateSkF}$ creates decision tree *t* using samples $\varSigma$ over the feature set. Lines 7–10 generate candidate Skolem function $\psi _j$ by iterating over all the leaf nodes of *t*. In particular, if a leaf node is labeled with 1, the candidate function is updated by disjoining with the formula returned by subroutine $\textsf {Path}$. $\textsf {CandidateSkF}$ also updates $d_j$ in *D*, $d_j$ is set of all *Y* variables on which, $y_j$ depends. If $y_j$ depends on $y_k$, then by transitivity $y_j$ also depends on $d_k$; in line 12, $\textsf {CandidateSkF}$ updates $d_j$ accordingly.

$\textsf {FindOrder}$: $\textsf {FindOrder}$ takes *D* as an input to output a valid linear extension of the partial order $\prec _{d}$ defined over $\{y_1, \ldots y_m\}$ with respect to the candidate Skolem function vector $\varPsi$.
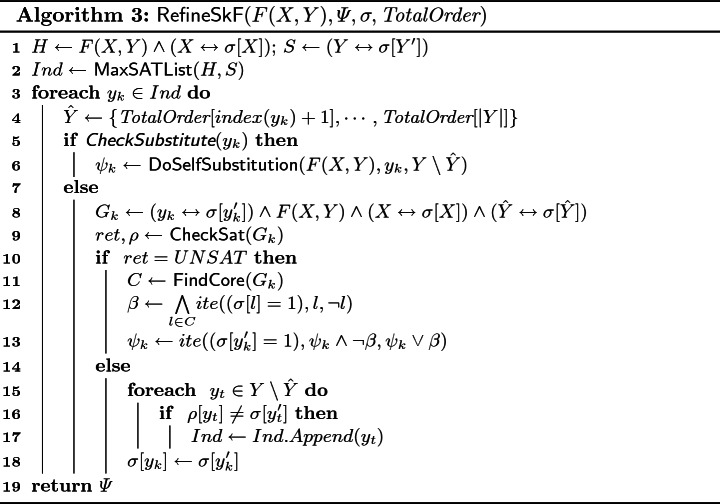


$\textsf {RefineSkF}$: $\textsf {RefineSkF}$ is invoked with a counterexample $\sigma$. $\textsf {RefineSkF}$ first performs *fault localization* to find the initial set of erring candidate functions; to this end, it calls the $\textsf {MaxSATList}$ subroutine (line 2) with $F(X,Y) \wedge (X \leftrightarrow \sigma [X])$ as hard-constraints and $(Y \leftrightarrow \sigma [Y])$ as soft-constraints. $\textsf {MaxSATList}$ employs a MaxSAT solver to find the solution that satisfies all the hard constraints and maximizes the number of satisfied soft constraints, and then returns a list (*Ind*) of *Y* variables such that for each of the variables appearing in (*Ind*) the corresponding soft-constraint was not satisfied by the optimal solution returned by MaxSAT solver.

Since candidate Skolem function corresponding to the variables in *Ind* needs to refine, $\textsf {RefineSkF}$ now attempts to synthesize a repair for each of these candidate Skolem functions. Repair synthesis loop (lines 3–19) starts off by collecting the set of *Y* variables, $\hat{Y}$, on which $y_k$ of *Ind* can depend on as per the ordering constraints (line 4). Next, it invokes the subroutine $\textsf {CheckSubstitute}$, which returns True if the candidate function corresponding to $y_k$ has been refined more than a chosen threshold times (fixed to 10 in our implementation), and the corresponding decision tree constructed during execution $\textsf {CandidateSkF}$ has exactly one node. If $\textsf {CheckSubstitute}$ returns true, $\textsf {RefineSkF}$ calls $\textsf {DoSelfSubstitution}$ to perform self-substitution. $\textsf {DoSelfSubstitution}$ takes a formula *F*(*X*, *Y*), an existentially quantified variable $y_k$ and a list of variables which depends on $y_k$ and performs self substitution of $y_k$ with constant 1 in the formula *F*(*X*, *Y*) [28].

If $\textsf {CheckSubstitute}$ returns false, $\textsf {RefineSkF}$ attempts a proof-guided repair for $y_k$. $\textsf {RefineSkF}$ calls $\textsf {CheckSat}$ in line 9 on $G_{k}$, which corresponds to formula 2: if $G_{k}$ is SAT, then $\textsf {CheckSat}$ returns a satisfying assignment$(\rho )$ of $G_{k}$ in $\sigma$, else $\textsf {CheckSat}$ returns unsatisfiable in the result, *ret*.

1. If *ret* is UNSAT, we proceed to refine $\psi _k$ such that for $\psi _k(X\mapsto \sigma [X],\hat{Y}\mapsto \sigma [\hat{Y}]) = \sigma [y_k]$. Ideally, we would like to apply a refinement that generalizes to potentially other counter-examples, i.e. solutions of $E(X,Y,Y')$. To this end, $\textsf {RefineSkF}$ calls $\textsf {FindCore}$ with $G_{k}$; $\textsf {FindCore}$ returns the list of variables (*C*) that occur in the clauses of UnsatCore of $G_{k}$. Accordingly, the algorithm constructs a *repair formula* $\beta$ as a conjunction of literals in $\sigma$ corresponding to variables in *C* (line 12). If $\sigma [y'_k]$ is 1, then $\psi _k$ is $\psi _k$ with conjunction of negation of $\beta$ and if $\sigma [y'_k]$ is 0, then $\psi _k$ is $\psi _k$ with disjunction of $\beta$.
2. If *ret* is SAT and $\rho$ is a satisfying assignment of $G_{k}$, then there exists a Skolem function vector such that the value of $\psi _k$ agrees with $\sigma [y_k]$ for the valuation of *X* and $\hat{Y}$ set to $\sigma [X]$ and $\sigma [\hat{Y}]$. However, for any $y_t \in Y \setminus \hat{Y}$ if $\sigma [y'_t] \ne \rho [y'_t]$, then for such a $y_t$, the Skolem function corresponding to $y_t$ may need to refine . Therefore, $\textsf {RefineSkF}$ adds $y_t$ to list of candidates to refine, *Ind*. Note that since $\sigma \models E(X,Y,Y')$, there exists at least one iteration of the loop (lines 3–18) where *ret* is UNSAT.

$\textsf {Substitute}$: To return the Skolem functions in terms of only *X*, $\textsf {Manthan}$ invokes $\textsf {Substitute}$ subroutine. For each $y_j$ of *Y* variable, $\textsf {Substitute}$ consider *Y* variables that occurs later in *TotalOrder* as $\hat{Y}$. Then, for each $y_i$ of $\hat{Y}$; it substitutes corresponding Skolem function $\psi _i$ in the Skolem function $\psi _j$ of $y_j$.

An example to illustrate our algorithm is deferred to the technical report [22].

## Experimental Results

We evaluate the performance of $\textsf {Manthan}$ on the union of all the benchmarks employed in the most recent works [4, 5],which includes 609 benchmarks from different sources: Prenex-2QBF track of QBFEval-17 [2], QBFEval-18 [3], disjunctive [6], arithmetic [45] and factorization [6]. We ran all the tools as per the specification laid out by their authors. We used Open-WBO [34] for our MaxSAT queries and PicoSAT [11] to compute UnsatCore. We used PicoSAT for its ease of usage and we expect further performance improvements by upgrading to one of the state of the art SAT solvers. We have used the Scikit-Learn [37] to create decision trees in $\textsf {LearnSkF}$ phase of $\textsf {Manthan}$. We have also used ABC [31] to represent and manipulate Boolean functions. To allow for the input formats supported by the different tools, we use the utility scripts available with the BFSS distribution [5] to convert each of the instances to both QDIMACS and Verilog formats. For $\textsf {Manthan}$, unless otherwise specified, we set the number of samples according to heuristic based on |*Y*| as described in Sect. 6.3 and minimum impurity decrease to 0.005. All our experiments were conducted on a high-performance computer cluster with each node consisting of a E5-2690 v3 CPU with 24 cores and 96 GB of RAM, with a memory limit set to 4 GB per core. All tools were run in a single-threaded mode on a single core with a timeout of 7200 s.

The objective of our experimental evaluation was two-fold: to understand the impact of various design choices on the runtime performance of $\textsf {Manthan}$ and to perform an extensive comparison of runtime performance vis-a-vis state of the art synthesis tools. In particular, we sought to answer the following questions:

1. How does the performance of $\textsf {Manthan}$ compare with state of the functional synthesis engines?
2. How do the usage of different sampling schemes and the quality of samplers impact the performance of $\textsf {Manthan}$?
3. What is the impact of $\textsf {LearnSkF}$ on the performance of $\textsf {Manthan}$?
4. What is the distribution of the time spent in each phase of $\textsf {Manthan}$?
5. How does using MaxSAT solver to identify the potential erring Skolem functions impacts on the performance of $\textsf {Manthan}$?
6. How does employing self-substitution for some Skolem functions impact $\textsf {Manthan}$?

We observe that $\textsf {Manthan}$ significantly improves upon state of the art, and solves 356 benchmarks while the state of the art tool can only solve 280; in particular, $\textsf {Manthan}$ solves 60 more benchmarks that could not be solved by any of the state of the art tools. To put the runtime performance statistics in a broader context, the number of benchmarks solved by techniques developed over the past five years range from 206 to 280, i.e., a difference of 74, which is same as an increase of 76 (i.e., from 280 to 356) due to $\textsf {Manthan}$.

Our experimental evaluation leads to interesting conclusions and several directions for future work. We observe that the performance of $\textsf {Manthan}$ is sensitive to different sampling schemes and the underlying samplers; in fact, we found that biased sampling yields better results than uniform sampling. This raises interesting questions on the possibility of designing specialized samplers for this task. Similarly, we observe interesting trade offs between the number of samples and the minimum impurity decrease in $\textsf {LearnSkF}$. The diversity of our extensive benchmark suite produces a nuanced picture with respect to time distribution across different phases, highlighting the critical nature of each of the phases to the performance of $\textsf {Manthan}$. $\textsf {Manthan}$ shows significant performance improvement by using MaxSAT solver to identify candidates to refine. $\textsf {Manthan}$ also has significant performance improvement with self substitution in terms of the required number of refinements.

### Comparison with Other Tools

We now present performance comparison of $\textsf {Manthan}$ with the current state of the art synthesis tools, BFSS [5], C2Syn [4], BaFSyn [14] and the current state of the art 2-QBF solvers CADET [39],CAQE [40] and DepQBF [32]. The certifying 2-QBF solver produces QBF certificates, that can be used to extract Skolem functions [8]. Developers of BaFSyn and DepQBF confirmed that the tools produce Skolem function for only valid instances, i.e. when $\forall X \exists Y F(X,Y)$ is valid. Note that the current version of CAQE does not support certification and we have used CAQE version 2 for the experiments after consultation with the developers of CAQE.

**Table 1.** No. of benchmarks solved by different tools

Total	BaFSyn	CAQE	DepQBF	C2Syn	BFSS	CADET	$\textsf {Manthan}$	All tools
609	13	54	59	206	247	280	$\textit{\textbf{356}}$	476

We present the number of instances solved Table 1. Out of 609 benchmarks, the most number of instances solved by any of the remaining techniques is 280 while $\textsf {Manthan}$ is able to solve 356 instances – a significant improvement over state of the art. We will focus on top 4 synthesis tools from Table 1 for further analysis.

**Fig. 2.**
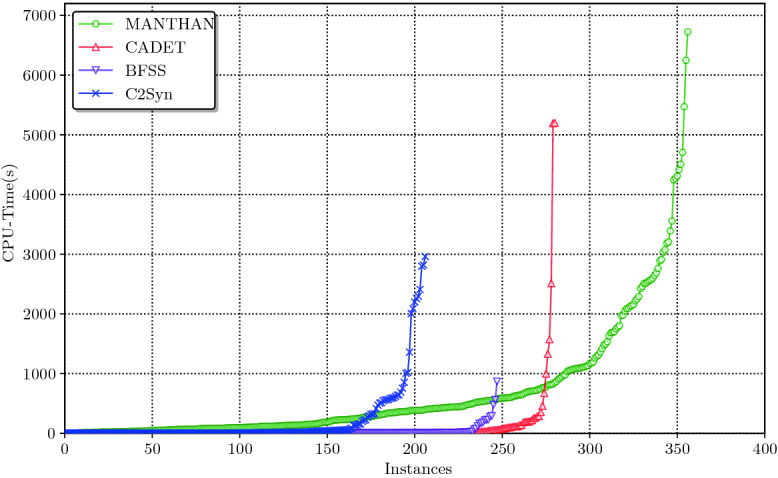
$\textsf {Manthan}$ versus competing tools for Skolem function synthesis

For a deeper analysis of runtime behavior, we present the cactus plot in Fig. 2: the number of instances are shown on the *x*-axis and the time taken on the *y*-axis; a point (*x*, *y*) implies that a solver took less than or equal to *y* seconds to find Skolem function of *x* instances on a total of 609 instances. An interesting behavior predicted by cactus plot and verified upon closer analysis is that for instances that can be solved by most of the tools, the initial overhead due to a multi-phase approach may lead to relatively larger runtime for $\textsf {Manthan}$. However, with the rise in empirically observed hardness of instances, one can observe the strengths of the multi-phase approach. Overall, $\textsf {Manthan}$ solves 76 more instances than the rest of the remaining techniques.

**Table 2.** Manthan vs other state-of-the-art tools

		C2Syn	BFSS	CADET	All tools
$\textsf {Manthan}$	Less	13	85	111	122
	More	$\textit{\textbf{163}}$	$\textit{\textbf{194}}$	$\textit{\textbf{187}}$	$\textit{\textbf{60}}$

We show a pairwise comparison of $\textsf {Manthan}$ vis-a-vis other techniques in Table 2. The second row of the table lists the number of instances that were solved by the technique in the corresponding column but not by $\textsf {Manthan}$ while the third row lists the number of instances that were solved by $\textsf {Manthan}$ but not the corresponding technique. First, we observe that $\textsf {Manthan}$ solves 163, 194, and 187 instances that are not solved by C2Syn, BFSS, and CADET respectively. Though BFSS and CADET solve more than 80 instances that $\textsf {Manthan}$ does not solve, they are not complementary; there are only 121 instances that can be solved by either BFSS or CADET but $\textsf {Manthan}$ fails to solve. A closer analysis of $\textsf {Manthan}$’s performance on these instances revealed that the decision trees generated by $\textsf {CandidateSkF}$ were shallow, which is usually a sign of significant under-fitting. On the other hand, there are 130 instances that $\textsf {Manthan}$ solves, but neither CADET nor BFSS can solve. These instances have high dependencies between variables that $\textsf {Manthan}$ can infer from the samples en route to predicting good candidate Skolem functions. Akshay et al. [4] suggest that C2Syn is an orthogonal approach to BFSS. $\textsf {Manthan}$ solves 81 instances that neither C2Syn nor BFSS is able to solve, and these tools together solve 86 instances that $\textsf {Manthan}$ fails to solve. Overall, $\textsf {Manthan}$ solves $\textit{\textbf{60}}$ instances beyond the reach of any of the above state of the art tools.

### Impact of the Sampling Scheme

To analyze the impact of the adaptive sampling and the quality of distributions generated by underlying samplers, we augmented $\textsf {Manthan}$ with samples drawn from different samplers for adaptive and non-adaptive sampling. In particular, we employed QuickSampler [16], KUS [42], UniGen2 [15], and BiasGen^1^. The samplers KUS and UniGen2 could only produce samples for mere 14 and 49 benchmarks respectively within a timeout of 3600 s. Hence, we have omitted KUS and UniGen2 from further analysis. We also experimented with a naive enumeration of solution using off-the-shelf SAT solver, CryptoMiniSat [43]. It is worth noting that QuickSampler performs worse than BiasGen for uniformity testing using Barbarik [13]. In our implementation, we had to turn off the validation phase of QuickSampler to allow generation number of samples within a reasonable time. To statistically validate our intuition described in Sect. 4, we performed adaptive sampling using BiasGen. We use $\textsf {AdaBiasGen}$ to refer to the adaptive sampling implementation.

Table 3 presents the performance of $\textsf {Manthan}$ with different samplers listed in Column 1. The columns 2, 3, and 4 lists the number of instances that were solved during the execution of respective phases: $\textsf {Preprocess}$, $\textsf {LearnSkF}$, and $\textsf {Refine}$. Finally, column 5 lists the total number of instances solved. Two important findings emerge from Table 3: Firstly, as the quality of samplers improve, so does the performance of $\textsf {Manthan}$. In particular, we observe that with the improvement in the quality of samples leads to $\textsf {Manthan}$ solving more instances in $\textsf {LearnSkF}$. Secondly, we see a significant increase in the number of instances that can be solved due to $\textsf {LearnSkF}$ with samples from $\textsf {AdaBiasGen}$. It is worth remarking that one should view the adaptive scheme proposed in Sect. 4 to be a proof of concept and our results will encourage the development of more complex schemes.

**Fig. 3.**
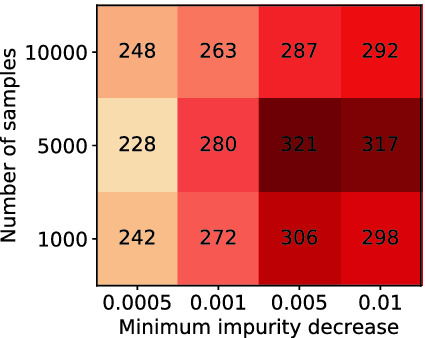
Heatmap of # instances solved. (Color figure online)

**Table 3.** $\textsf {Manthan}$ with different samplers

Sampler	No. of instances solved			#Solved
	$\textsf {Preprocess}$	$\textsf {LearnSkF}$	$\textsf {Refine}$	
CryptoMiniSat	66	14	191	271
QuickSampler	66	28	181	275
BiasGen	66	51	228	345
$\textsf {AdaBiasGen}$	66	66	224	$\textit{\textbf{356}}$

### Impact of $\textsf {LearnSkF}$

To analyze the impact of different design choices in $\textsf {LearnSkF}$, we analyzed the performance of $\textsf {Manthan}$ for different samples (1000, 5000 and 10000) generated by $\textsf {GetSamples}$ and for different choices of minimum impurity decrease (0.001, 0.005, 0.0005). Figure 3 shows a heatmap on the number of instances solved on each combination of the hyperparameters; the closer the color of a cell is to the red end of the spectrum, the better the performance of $\textsf {Manthan}$.

At the first look, Fig. 3 presents a puzzling picture: It seems that increasing the number of samples does not improve the performance of $\textsf {Manthan}$. On a closer analysis, we found that the increase in the number of samples leads to an increase in the runtime of $\textsf {CandidateSkF}$ but without significantly increasing the number of instances solved during $\textsf {LearnSkF}$. The runtime of $\textsf {CandidateSkF}$ is dependent on the number of samples and |*Y*|. On the other hand, we see an interesting trend with respect to minimum impurity decrease where the performance first improves and then degrades. A plausible explanation for such a behavior is that with an increase in *minimum impurity decrease*, the generated decision trees tend to underfit while significantly low values of *minimum impurity decrease* lead to overfitting. We intend to study this in detail in the future.

Based on the above observations, we set the value of minimum impurity decrease to 0.005 and set the number of samples to (1) 10000 for $|Y| < 1200$, (2) 5000 for $1200 < |Y| \le 4000$, and (3) 1000 for $|Y| > 4000$.

### Division of Time Taken Across Different Phases

**Fig. 4.**
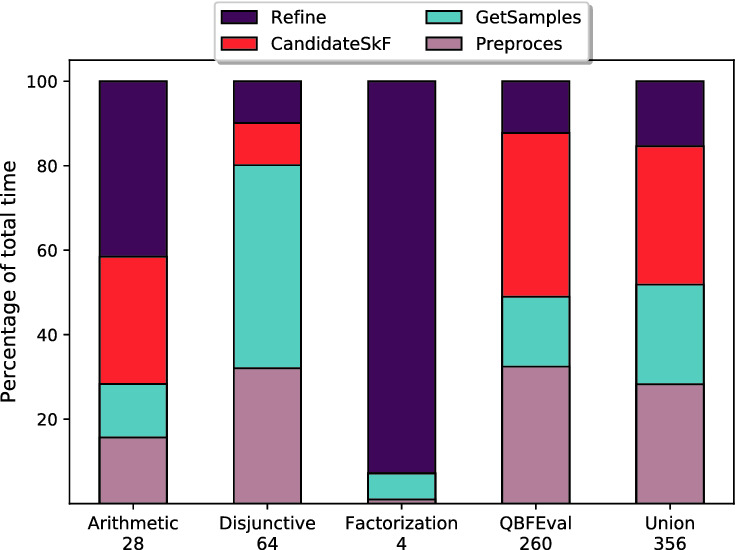
Fraction of time spent in different phases in $\textsf {Manthan}$ over different classes of benchmarks. (Color figure online)

To analyze the time taken by different phases of $\textsf {Manthan}$ across different categories of the benchmarks, we normalize the time taken for each of the four core subroutines, $\textsf {Preprocess}$, $\textsf {GetSamples}$, $\textsf {CandidateSkF}$, and $\textsf {RefineSkF}$, for every benchmark that was solved by $\textsf {Manthan}$ such that the sum of time taken for each benchmark is 1. We then compute the mean of the normalized times across different categories instances. Figure 4 shows the distribution of mean normalized times for different categories: Arithmetic, Disjunction, Factorization, QBFEval, and all the instances.

The diversity of our benchmark suite shows a nuanced picture and shows that the time taken by different phases strongly depends on the family of instances. For example, the disjunctive instances are particularly hard to sample and an improvement in the sampling techniques would lead to significant performance gains. On the other hand, a significant fraction of runtime is spent in the $\textsf {CandidateSkF}$ subroutine indicating the potential gains due to improvement in decision tree generation routines. In all, Fig. 4 identifies the categories of instances that would benefit from algorithmic and engineering improvements in $\textsf {Manthan}$’s different subroutines.

### Impact of Using MaxSAT

In $\textsf {RefineSkF}$, $\textsf {Manthan}$ invokes the $\textsf {MaxSATList}$ subroutine, which calls MaxSAT solver to identify the potential erring Skolem functions. To observe the impact of using MaxSAT solver to identify the candidates to refine, we did an experiment with $\textsf {Manthan}$, without $\textsf {MaxSATList}$ subroutine call. For all $y_i$, where $\sigma [y_i] \ne \sigma [y'_i]$ were considered as candidates to refine. $\textsf {Manthan}$ without $\textsf {MaxSATList}$ subroutine call solved 204 instances that represents a significant drop in the number of solved instances by $\textsf {Manthan}$ with $\textsf {MaxSATList}$ subroutine.

### Impact of Self-substitution

**Fig. 5.**
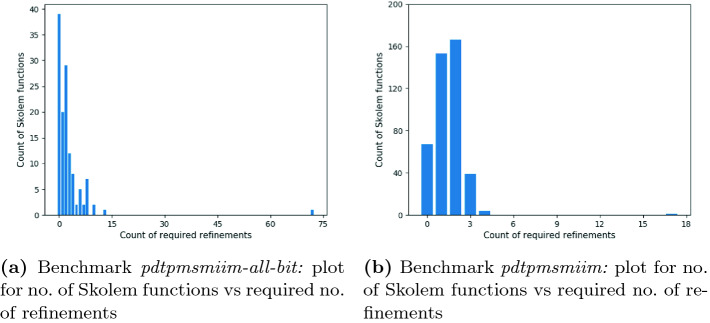
The plots to show the required number of refinements for the candidate Skolem functions.

To understand the impact of self-substitution, we profile the behavior of candidate Skolem functions with respect to number of refinements for two of our benchmarks; *pdtpmsmiim-all-bit* and *pdtpmsmiim*. In Fig. 5, we use histograms with the number of candidate Skolem functions on y-axis and required number of refinements on x-axis. A bar of height *a* i.e $y=a$ at *b* i.e $x=b$ in Fig. 5 represents that *a* candidate Skolem functions converged in *b* refinements. The histograms show that only a few Skolem functions require a large number of refinements: the tiny bar towards the right end in Fig. 5a represents that for the benchmark *pdtpmsmiim-all-bit* only 1 candidate Skolem function required more than 60 refinements whereas all other candidate Skolem functions needed less than 15 refinements. Similarly, for the benchmark *pdtpmsmiim*, Fig. 5b shows that only 1 candidate Skolem function was refined more than 15 times, whereas all other Skolem functions required less than 5 refinements. We found similar behaviors in many of our other benchmarks.

Based on the above trend and an examination of the decision trees corresponding to these instances, we hypothesize that some Skolem functions are hard to learn through data. For such functions, the candidate Skolem function generated from the data-driven phase in $\textsf {Manthan}$ tends to be poor, and hence $\textsf {Manthan}$ requires a long series of refinements for convergence. Since our refinement algorithm is designed for small, efficient corrections, we handle such hard to learn Skolem functions by synthesizing via self-substitution. $\textsf {Manthan}$ detects such functions via a threshold on the number of refinements, which is empirically determined as 10, to identify hard to learn instances and sets them up for self-substitution.

In our experiments, we found 75 instances out of 356 solved instances required self-substitution, and for 51 of these 75 instances, only one variable undergoes self-substitution. Table 4 shows the impact of self-substitution for five of our benchmarks: $\textsf {Manthan}$ has significant performance improvement with self-substitution in terms of the required number of refinements, which in turns affects the overall time. Note that $\textsf {Manthan}$ can refine multiple candidates in a single $\textsf {RefineSkF}$ call. For the first four benchmarks, all the other Skolem function except the poor candidates were synthesized earlier than 10 refinement iteration, and at the $10^{th}$ refinement iteration the poor candidate functions hit our threshold for self-substitution. Taking the case of the last benchmark, all the other Skolem functions for it were synthesized earlier than 40 refinement cycles, and the last 16 iterations were only needed for 2 of the poor candidate functions to hit our threshold for self-substitution. Note that self-substitution can lead to an exponential blowup in the size of the formula, but it works quite well in our design as most Skolem functions are learnt quite well in the $\textsf {LearnSkF}$ phase.

**Table 4.** $\textsf {Manthan}$ : Impact of self substitution

Benchmarks $\exists Y F(X,Y)$	|*X*|	|*Y*|	No. of refinements		Time(s)	
			Self-substitution		Self-substitution	
			Without	With	Without	With
kenflashpo2-all-bit	71	32	319	10	35.88	19.22
eijkbs1512	316	29	264	10	42.88	32.35
pdtpmsmiim-all-bit	429	30	313	10	72.75	36.08
pdtpmssfeistel	1510	68	741	10	184.11	115.07
pdtpmsmiim	418	337	127	56	1049.29	711.48

## Conclusion

Boolean functional synthesis is a fundamental problem in Computer Science with a wide variety of applications. In this work, we propose a novel data-driven approach to synthesis that employs constrained sampling techniques for generation of data, machine learning for candidate Skolem functions, and automated reasoning to verify and refine to generate Skolem functions. Our approach achieves significant performance improvements. As pointed out in Sects. 5 and 6, our work opens up several interesting directions for future work at the intersection of machine learning, constrained sampling, and automated reasoning.

## Footnotes

1 BiasGen is developed by Mate Soos and Kuldeep S. Meel, and is pending publication.
